# The Principles and Practice of Dental Surgery

**Published:** 1845-05

**Authors:** 


*The Principles and Practice of Dental Surgery.* By Chapin A. Harris, M. D., D. D. S.; Professor of Practical Dentistry and Dental Pathology in the Baltimore College of Dental Surgery; Fellow of the American Society of Dental Surgeons; Member of the Medico-Chirurgical Faculty of Maryland, etc. etc. Second Edition. Revised, modified, and greatly enlarged. Illustrated by sixty-nine wood engravings. 8vo. pp. 600. Philadelphia. Lindsay & Blakiston. 1845.

The first edition of this excellent work was so much lauded at the time of its publication, by experienced dentists, and has met with such ready sale, that it seems scarcely necessary to speak now of its general merits. As may well be supposed, a second edition by so competent an author, possesses many advantages over the first. Several very important subjects, not embraced in the former edition, are treated of in this; such as the Anatomy and Physiology of the Mouth; the Physical Characteristics of the Teeth and Gums; Salivary Calculus; Diseases of the Maxillary Sinus, &c. These are useful and important additions. No work on Dentistry can, indeed, be regarded as complete, which does not contain an account of the anatomy and physiology of the mouth, including the teeth and gums.

"The object of the author in the preparation of this edition has been," as we are informed, "to provide a thorough elementary treatise on Dental Medicine and Surgery, which might be a text book for the student, and a guide for the more inexperienced practitioner," and we think it will be conceded by all who examine the work, that he has succeeded in attaining the end at which he has aimed. We can hardly doubt that every dentist in the country will supply himself with a copy of Dr. Harris' work; and we hope that country practitioners of medicine, too, will avail themselves of the information and excellent advice it contains. They are often called on to treat diseases of the teeth and their consequences, and have need, therefore, of more instruction on these points than is generally conveyed in medical lectures, and the books ordinarily in the hands of physicians.

The style in which the book is got up is exceedingly creditable to the publishers—substantial binding, good paper, clear type, and excellent wood engravings, all add to its value.

